# Denial of Earned Wages and Psychological Distress among Latino Day Laborers: The Impact of Work-Related Mistreatment and Powerlessness on Mental Health

**DOI:** 10.1007/s40615-026-02972-6

**Published:** 2026-04-29

**Authors:** Lynn N. Ibekwe-Agunanna, John S. Atkinson, Nanjun Chen, Yesmel A. King, Maria Eugenia Fernández-Esquer

**Affiliations:** 1 O’Donnell School of Public Health, The University of Texas Southwestern Medical Center, Dallas, TX, USA; 2 Harold C. Simmons Comprehensive Cancer Center, The University of Texas Southwestern Medical Center, Dallas, TX, USA; 3 School of Public Health, The University of Texas Health Science Center at Houston, Houston, TX, USA; 4 Center for Health Promotion and Prevention Research, The University of Texas Health Science Center at Houston, Houston, TX, USA

**Keywords:** Latino immigrants, Day laborer, Unpaid or underpaid labor, Mental health, Powerlessness, Structural vulnerability

## Abstract

Intentional failure to pay workers their full wage for completed work—either by not paying at all or paying less than promised—is a widespread form of workplace abuse that disproportionately affects low-wage immigrant workers. Latino day laborers (LDLs) frequently experience this mistreatment, which can undermine their financial stability and mental health. In this study, we examined associations between being denied earned wages (not paid at all or paid less than promised) and select indicators of psychological distress (depression, state anxiety, and perceived stress). We also explored the influence of perceived powerlessness on these associations. A cross-sectional survey was conducted with 300 LDLs at 18 day-labor hiring sites. Participants reported the number of times in the past month they had (1) not been paid at all and (2) been paid less than promised for a completed job. Perceived powerlessness, depression, anxiety, and stress were assessed using validated measures. Linear regression was used to estimate associations between each wage denial exposure and each psychological distress indicator, adjusted for sociodemographic characteristics. Path analyses were conducted to evaluate whether powerlessness accounted for associations between wage denial and psychological distress. Regressions revealed that being paid less than promised was associated with greater depression, anxiety, and stress. Furthermore, path analyses indicated that powerlessness may partially account for 36.1% of the observed association with depression, 16.2% with anxiety, and 27.7% with stress. These findings point to being denied earned wages for completed work as an important contributor to Latino day laborers’ vulnerability to psychological distress.

## Introduction

The intentional *nonpayment* and *underpayment* of wages due from an employer to a worker(s) for a job that has been completed is a labor law violation and major public health issue in the United States (U.S.) [1–3]. This form of workplace abuse is widespread in the U.S. labor market and a significant contributor to low wages and low-income status among workers, disproportionately affecting immigrant workers and other marginalized groups in low-wage jobs [1–6]. The consequences are far-reaching, impacting workers’ ability to pay for basic necessities, including health care, and may also impact workers’ physical and mental health [7–12].

Being denied earned wages for completed work has previously been conceptualized as the direct result of interconnected systemic factors that disadvantage specific groups, including immigrant and low-wage workers [3, 12–15]. These factors include historical and contemporary labor practices, gaps in legal protections, and prevailing societal narratives that devalue immigrant labor, particularly for individuals with limited legal protections [2, 8, 16–21]. Together, these conditions create an environment in which being denied due wages for completed work is more likely to occur, as well as remain unreported or unremedied [6, 12].

Although difficult to estimate accurately, non/underpayment of earned wages is estimated to cost workers at least $8 billion annually [2]. It is estimated that unpaid wages for completed work may represent up to 25% of a worker’s income, and the decrease in wages may lead to workers’ deprivation, chronic stress, loss of self-esteem, and diminished social status [2, 11]. Although additional research is needed, studies indicate that preventing non/underpayment of earned wages may reduce occupational and income inequalities and increase workers’ life expectancy [9, 11].

For *Latino day laborers (LDLs)*—a particularly vulnerable group of workers who regularly face workplace discrimination, abuse, and exploitation—the denial of payment for completed work is one of the most pervasive and prominent forms of workplace abuse they experience [6, 8, 16, 22–24]. Day labor is a type of informal and temporary employment characterized by a daily search for work, and is often associated with low and unstable wages, insecure work, high rates of workplace injury, and recurrent exposure to labor violations such as being denied earned wages [22, 25, 26]. These labor conditions place day laborers at heightened risk of financial strain and psychological distress [22, 27, 28].

Latino immigrants constitute the majority of the day labor workforce in the U.S., with a large percentage coming from Mexico and Central America [22]. Research over the last two decades documents high rates of LDLs not being paid their due wages for completed work (reported by LDLs in research/non-law enforcement contexts), ranging across studies from 24.5% to 80% depending on study factors such as study location, design, and measures [8, 12, 16, 22, 29–31]. However, the true prevalence of this type of workplace abuse is likely higher given that LDLs often do not report these incidents to authorities due to fear of deportation and a general distrust of law enforcement [8, 27, 28, 30, 31].

While existing laws in the U.S. offer protection against worker mistreatment regardless of immigration status, the inconsistent enforcement of these laws enables the continuation of these practices [2, 18–20]. This gap in enforcement is further exacerbated by labor and immigration policies that marginalize immigrant workers and contribute to public narratives questioning their entitlement to legal protections [4, 10, 18, 32, 33]. Employers who withhold wages often act with impunity, taking advantage of lax labor law enforcement, the real or perceived immigration status of LDLs, and prevailing anti-immigrant sentiments [2, 4, 6, 8, 10, 16–20, 32, 33]. Additionally, factors such as limited education, English proficiency, and knowledge of labor laws increase LDLs’ vulnerability to being denied their due wages [8, 28].

Denying a worker’s due wages for completed work is not only a labor and economic issue; it is also a significant social and personal stressor that can lead to serious mental health consequences. According to qualitative research, LDLs have frequently described these experiences as demoralizing, mentally taxing, and a source of significant stress. This can contribute to feelings of worthlessness, depression, anxiety [27, 28, 34–36], and *desesperación*—a culturally specific idiom for psychological distress [28, 36]. While these studies suggest a connection between experiencing total nonpayment or underpayment of earned wages for completed work and LDLs mental health, there is limited quantitative research that directly examines this relationship.

To our knowledge, only three studies have quantitatively examined this relationship within the Latino day laborer population; of which, one reported null findings (likely due to measurement challenges) [12], and the remaining found that being denied earned wages was significantly associated with depression and post-traumatic stress disorder [26, 31]. Broader quantitative studies in other populations, while also limited, provide additional support [10, 37]. Using longitudinal data from a nationally representative cohort, Prins et al. found that higher unconcealed exploitation (a measure of earned labor income not being paid to workers) was associated with increased psychological distress and substantially higher odds of mental illness [37]. Further supporting this pattern is population-based survey analyses conducted by Lee and colleagues, which indicated that being denied due wages (operationalized as not being paid for hours worked) was significantly associated with increased psychological distress, as well as poorer self-rated health, among immigrant and low-wage workers [10].

Each of the studies referenced above are an important contribution to the day laborer/worker health literature, suggesting that being denied earned wages represents a salient factor associated with worker’s mental health. However, there are notable limitations: they either report null findings [12]; utilize index measures that include but do not isolate being denied earned wages as a distinct variable [27]; or examine broader immigrant or low-wage worker populations rather than day laborers specifically [32]. These gaps highlight the need for direct quantitative research that explicitly measures experiences of being denied earned wages and tests their association with adverse mental health outcomes among LDLs.

Although quantitative evidence directly linking this type of work-related mistreatment to adverse mental health outcomes is limited, evidence from the broader day labor, worker health, and low-wage/minimum wage/income policy literature supports this conceptual framework. For example, chronically low and unstable wages/income (conceptually related to not being paid earned wages in that both restrict material resources, heighten economic insecurity, and reduce workers’ sense of control) are consistently reported as key contributors to poor mental health outcomes such as psychological distress, depressive symptoms, anxiety, and suicidality [9, 11, 37]. Relatedly, increases in minimum wages are associated with improvements in these outcomes [38, 39].

Additionally, literature on discrimination and psychological trauma offers insight into the mental health consequences of repeated mistreatment occurring within unequal power relations. Scholars have proposed frameworks to describe how persistent mistreatment based on social group membership can be a severe interpersonal stressor that contributes to psychological distress and fear [40–42]. For example, Chavez-Duenas et al. [41] used the term *ethno-racial trauma* to characterize emotional harm resulting from repeated exposure to exclusion, bias, and marginalization. These frameworks expand the traditional understanding of trauma, which has been limited to physical harm or threats, to include psychological and emotional injuries stemming from persistent social and structural mistreatment. This form of trauma is a chronic life stressor that can lead to feelings of increased vulnerability and impair everyday functioning, particularly among immigrant populations [42]. Exposure to such mistreatment can lead to intrusive thoughts, hypervigilance, avoidance, external locus of control, dissociation, and a sense of hopelessness [40, 42–44], while persistent social or identity-based mistreatment is a risk factor for developing PTSD and other mental health-related concerns, including anxiety, depression, suicidal ideation, social isolation, and maladaptive drinking behaviors [27, 34, 43, 45–49]. This is particularly concerning given that Latinos who report such experiences are more likely to conceal them after victimization and less likely to seek mental health services [42, 50].

In the day labor literature, high levels of stress [26, 27, 34, 51–56] as well as *allostatic load*—which is a measure of the cumulative physiological effects of chronic stress on the body over the life course [26, 57]—have been documented among LDLs due to various stressors. These include not being paid their full wage for completed work, employer abuse, dangerous working conditions, chronic employment insecurity, immigration-related worry, and adaptation stress. Among these, financial hardship and the income instability inherent in day labor work are consistently documented as the most salient stressors for LDLs, both pre- and post-immigration. This underscores the importance and need of quantitative research investigating the impact of being denied earned wages on mental health in this vulnerable population; and understanding the pathways through which this occurs will be equally important, providing valuable information that can inform intervention development.

*Perceived powerlessness*—or the extent to which an individual feels a lack of control over events affecting their personal life—may represent a salient psychosocial mechanism linking wage denial to psychological distress. Qualitative reports suggest that being denied earned wages for completed work can lead to a sense of powerlessness among LDLs due to a combination of individual vulnerabilities and structural conditions [27, 28, 34–36]. This sense of powerlessness is compounded by the precarious financial situations, cultural expectations, and immigration-related vulnerabilities many LDLs face, creating a cycle of exploitation and distress. For example, LDLs often have financial obligations both in the U.S. and in their home countries [31, 34, 51, 58], yet they have unpredictable and brief work as day laborers and often do not earn enough money to cover their basic living expenses and meet their additional financial commitments [54, 56]. Being denied their due wages directly undermines their ability to meet these obligations, exacerbating this lack of control over meeting their basic needs and providing for their families, which can take a significant psychological toll [27, 28, 34–36].

Notably, the vast majority of day laborers are men [22], and culturally, the role of provider is central to many Latino men’s identity and self-worth [27, 28, 34, 51, 59, 60]; thus, not being paid or being underpaid for completed work can be especially distressing and traumatic. Furthermore, for workers with uncertain or limited legal protections, fear of deportation may prevent them from reporting their victimization—further compounding their sense of powerlessness and increasing the risk of repeated victimization [8, 18, 27, 28, 30, 31]. This combination of economic exploitation, perceived powerlessness, and work-related mistreatment likely strains their already depleted mental resources, leading to even greater psychological distress.

Drawing on these insights, the goal of the current study was to estimate the association between two forms of being denied due wages for completed work—not being paid at all (total nonpayment) and being paid less than promised (underpayment)—and three indicators of psychological distress (depression, state anxiety, and stress) among Latino day laborers. Additionally, we explored whether perceived powerlessness was associated with these relationships. Through path analyses, we estimated the direction and magnitude of both direct and indirect associations. We hypothesized that not being paid at all and being paid less than promised would be positively associated with increased depression, anxiety, and stress, and that perceived powerlessness would mediate these associations.

## Methods

### Recruitment

Data for the current study were collected as part of a rapid needs assessment survey conducted among Latino day laborers between November and December 2021. The survey aimed to examine LDLs’ work-related experiences during the COVID-19 pandemic. Participants were recruited in the Houston metropolitan area from locations where LDLs commonly gather to seek work (e.g., parking lots of home improvement stores, convenience stores, gas stations, apartment complexes, public parks, and street intersections). These locations are referred to collectively as “corners.” Prior to recruitment, a list of 30 previously identified corners was randomly ordered. Corners at which 1 – 15 LDLs had been previously observed were classified as small. Corners with 16 – 30 LDLs were classified as medium, and corners with more than 30 LDLs were classified as large. For small and medium-sized corners, recruitment quotas were set at 80 participants for each size stratum. For large corners, a recruitment quota of 140 was set. The field team was instructed to recruit as many participants as possible during each corner visit and to move on to the next corner on the randomized list until the target enrollment for each corner size group was reached.

Participants were eligible if they were 18 years of age or older, self-identified as Latino, present at the corner seeking work, and had previously sought work at a corner. Trained, bilingual interviewers explained the purpose of the study, determined LDLs’ eligibility, and consented eligible candidates. Surveys developed in Spanish and programmed in Qualtrics were administered orally by trained interviewers using iPads, which took about 90 minutes to complete. Given the length of the survey and the time taken away from securing a job for the day, participants were compensated $75 for their time. Study procedures were approved by the Committee for the Protection of Human Subjects Institutional Review Board at The University of Texas Health Science Center at Houston.

A total of 416 LDLs were observed, of which 314 were approached. Of these, 304 provided verbal consent and 300 surveys were completed. Recruitment goals were met for each size stratum, with 80 surveys from small corners, 80 from medium corners, and 140 from large corners. A total of 18 corners were visited (6 small, 6 medium, and 6 large).

### Measures

#### Denied Earned Wages

The primary exposure of interest was having been **denied their due wages for a job they completed**, measured at two levels. Participants were asked to indicate the number of times in the past month they were: **(1) *not paid at all*** *(total nonpayment)*, and **(2) *paid less than promised*** *(underpayment)* after completing a job.

#### Perceived Powerlessness

The proposed mediator was **perceived powerlessness**, assessed using the nine-item **Perceived Powerlessness Scale (PPS)** developed by Lim, Powell, Xue et al. [61]. Sample item: “Sometimes you feel that you are being pushed around in life.” Responses were recorded on a four-point scale (1 = Strongly disagree; 2 = Disagree; 3 = Agree; and 4 = Strongly agree). Total PPS scores were computed by summing item responses, with higher scores reflecting greater endorsement of feelings of powerlessness (possible range: 9 – 36); total PPS scores were calculated only for participants with complete responses across all items. Cronbach’s α for the scale was .79.

#### Indicators of Psychological Distress

The outcome of interest was **psychological distress**, conceptualized as “an umbrella term encompassing multiple common psychological conditions, ranging from subclinical symptoms to clinical diagnoses of depression, anxiety, stress, or posttraumatic stress disorder (PTSD)” [62]. Thus, we examined three indicators of psychological distress: (1) depression, (2) state anxiety, and (3) stress, each measured using previously validated scales.

**Depression** was measured with the seven-item **Center for Epidemiologic Studies Depression Scale (CES-D)** [63]. Sample item: “In the last week, how often would you say you didn’t feel like eating; your appetite was poor?” Responses to each item of the scale were recorded on a four-point scale (1 = Not at all [< 1 day]; 2 = A little [1 – 2 days]; 3 = Frequently [3 – 4 days]; 4 = A lot [5 – 7 days]. Cronbach’s α for the scale was .87.

**State anxiety** was measured using the six-item **State-Trait Anxiety Inventory (STAI)** [64]. Sample item: “In general today, you feel nervous?” Three items reflecting a positive state (e.g., “calm”) were reverse scored. Responses were recorded on a four-point scale (1 = Not at all; 2 = Somewhat; 3 = Moderately so; and 4 = Very much so). Cronbach’s α for the scale was .73.

**Perceived Stress** was measured using six items from the 14-item **Perceived Stress Scale (PSS)** [65]. Sample item: “In the past 30 days how often have you been upset because of something that happened unexpectedly?” Responses were recorded on a five-point scale (1 = Never; 2 = Almost never; 3 = Sometimes; 4 = Fairly often; and 5 = Very often). Cronbach’s α for the scale was .88. Using the same approach as for the PPS, total scale scores were computed for each outcome measure (CES-D, STAI, and PSS), with higher scores reflecting greater endorsement of feelings corresponding to the outcome assessed (possible ranges: 7 – 28 for CES-D, 6 – 24 for STAI, and 6 – 30 for PSS).

#### Sociodemographic Characteristics

To characterize the sample, we measured age (computed as the number of days between the survey date and reported date of birth divided by 365.25); time in the United States (computed as the sum of reported years and months, with months divided by 12), years of school completed; marital status (single, never married, married or living with a partner, divorced, widowed, or separated), and the number of days in the last month they found work at the corner.

### Analyses

Of the 300 surveys collected, 290 had non-missing responses across all sociodemographic variables, and 274 had complete data across all exposure and outcome variables of interest; these data were used for analyses. For analytic purposes, marital status and both wage denial variables were recoded. Marital status was recoded as single/never married, married/living with a partner, or formerly married (divorced, widowed, or separated). The majority of participants reported that they had not been paid at all or had been paid less than promised four or fewer times in the past month; specifically, only four participants (1.4%) reported more than four instances of not being paid at all (total nonpayment), and only seven (2.4%) reported more than four instances of being paid less than promised (underpayment). Given the left-skewed distribution of these wage denial measures, all reports of more than four instances of non/underpayment were recoded as four instances.

Descriptive statistics, including means and standard deviations (sd) for continuous variables and frequencies for categorical variables, were computed for all study variables. Sample characteristics were further examined by dichotomizing each wage denial variable (0 vs. ≥1 instance) and comparing characteristics across these groups. Bivariate differences were assessed using t-tests for continuous measures, and chi-square or Fisher’s exact tests for categorical measures.

**Fig. 1:**
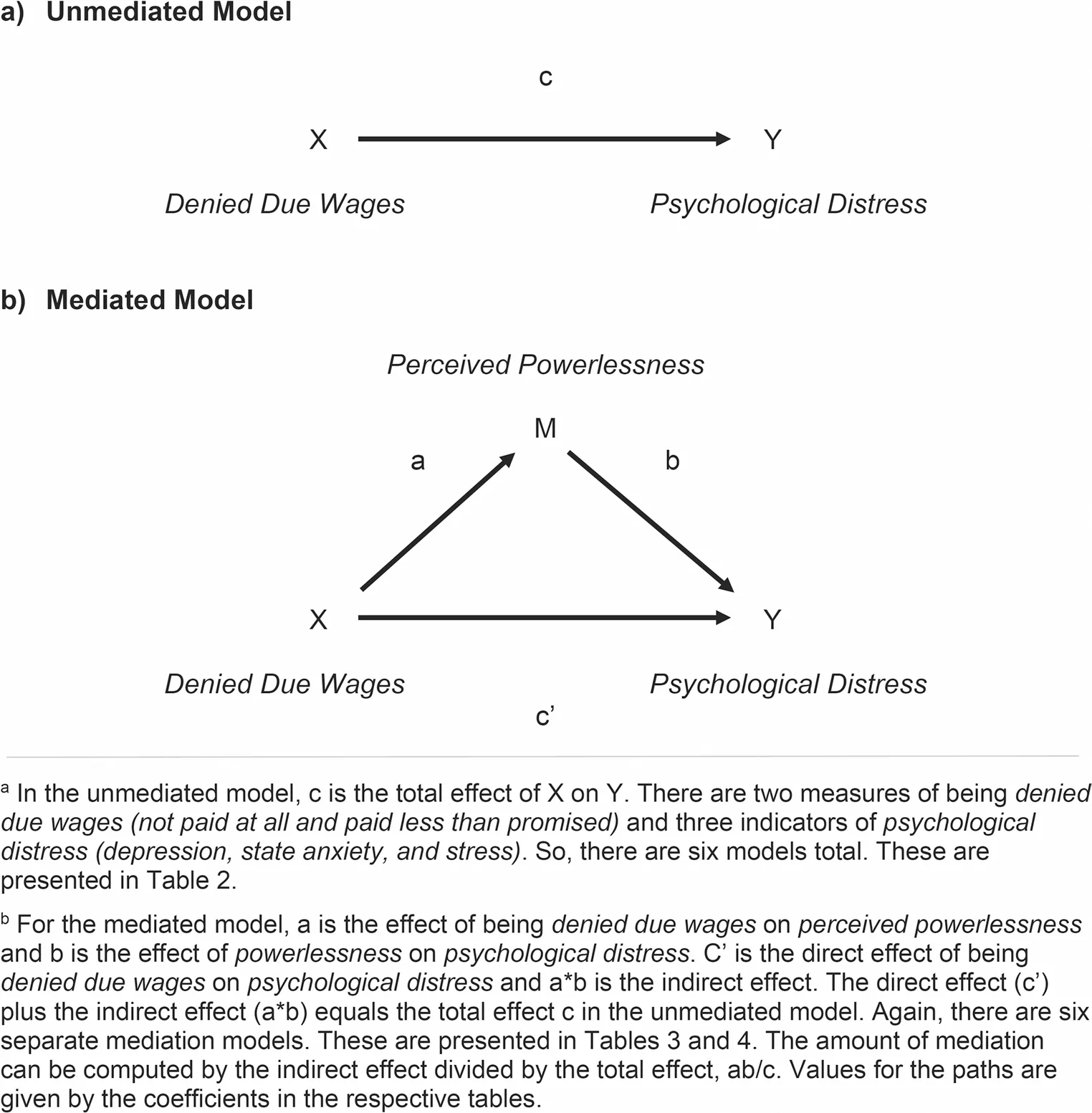
Conceptual Models for the Association Between Denied Wages and Psychological Distress, with and without Mediation by Perceived Powerlessness: (**A**) Unmediated model estimating the total association between denied due wages and psychological distress; (**B**) Mediated model estimating the indirect and direct effects of denied due wages on psychological distress through perceived powerlessness

The primary aim was to estimate associations between not being paid earned wages for completed work (total nonpayment or underpayment) and psychological distress indicators (depression, anxiety, stress), with a secondary aim of assessing whether perceived powerlessness mediated these associations. Our model of interest is presented in Figure 1 (panels A and 1B).

To assess the model, we applied the product of coefficients approach [66]. Unstandardized coefficients for the paths in the figures were derived from three regression equations:

(1) $$Y=b_{10}+b_{11}X+b_{12n}Z+e_{1}$$

(2) $$M=b_{20}+b_{21}X+b_{23n}Z+e_{2}$$

(3) $$Y=b_{30}+b_{31}X+b_{32}M+b_{33n}Z+e_{3}$$

Equations 1 – 3 were evaluated for each of the six possible combinations between our two exposures of interest and our three indicators of psychological distress. In these equations, b,b, andb represent the constant term (intercept), and e, e, and e represent the error terms. Z represents the set of demographic covariates, with each b representing the coefficient for the n covariate.

The coefficient b in Equation 1 represents the association between the given wage denial exposure (X) and the given psychological distress outcome (Y) (path c). This is the of not being paid due wages on an indicator of psychological distress (disregarding powerlessness). This addresses our primary aim.

The coefficient b in Equation 2 gives the association of the given wage denial measure (X) with powerlessness (M) (path a). The coefficient b in Equation 3 gives the association of powerlessness (M) with an indicator of psychological distress (Y), controlling for wage denial (M) (path b). The coefficient for b in Equation 3 gives the association of the given wage denial measure (X) with an indicator of psychological distress, controlling for powerlessness (M) (path c’).

The coefficient for c’ represents the *direct effect* between being denied due wages on indicators of psychological distress. The product of a*b constitutes the *indirect effect* of being denied due wages on the indicator of psychological distress via its effect on powerlessness. If this value is statistically significant, then there is a significant mediation effect; this addresses our secondary aim. The sum of the *direct effect* and the *indirect effect* is equal to the *total effect*. The amount of the *mediated effect* can be computed by the *indirect effect divided by the total effect (ab/c)*. Although results are presented in terms of “effects,” the data are cross-sectional; observed associations should not be interpreted as causal.

All statistical analyses were conducted using R (version 4.5). Path models were analyzed via Structural Equation Modeling, using lavaan package [67]. Regression coefficients (b), 95% confidence intervals (CI), and p-values were computed for each path. A significance level of p < .05, two-tailed, was used for all analyses. For each regression, sociodemographic variables (age, time in the US, years of school, marital status, and days found work) were entered as covariates.

Prior to conducting the mediation analyses, we evaluated linear model assumptions for each outcome using residual diagnostics. Visual inspection of Q-Q plots and results from the Shapiro-Wilk test indicated departures from normality and evidence of heteroscedasticity. Accordingly, we utilized non-parametric bootstrapping with 1,000 resamples to estimate robust standard errors and bias-corrected and accelerated (BCa) 95% confidence intervals for all direct and indirect effects. This approach provides a more accurate and stable test of the mediation pathways given the skewed distribution of our psychological distress outcomes and avoids interpretability losses associated with outcome transformation or dichotomization.

## Result

### Descriptive Statistics

Sample characteristics are shown in Table 1. All participants were male (not shown in the table) and, on average, were in their mid-40s (years of age), had lived in the United States for approximately 13 years, and had completed approximately eight years of schooling. In addition, nearly half of the sample (48.5%, n=133) were single or had never been married; 36.9% (n=101) were married or living with a partner; and 14.6% (n=40) were formerly married.

Overall, 14.6% of participants (n = 40) reported not being paid at all for a completed job (total nonpayment) at least once in the past month; the number of occurrences ranged from 0 to 4, with a mean of 0.3 (sd = 0.7). In addition, 28.5% of participants (n = 78) reported being paid less than promised (underpayment) at least once in the past month; the reported number of occurrences ranged from 0 to 4, with a mean of 0.6 (sd = 1.2). Overall, the mean total scale scores (mean of participants’ summed scale scores) were 22.6 (sd = 4.3) for perceived powerlessness (9-item PPS), 13.1 (sd = 4.9) for depression (7-item CES-D), 10.5 (sd = 3.4) for state anxiety (6-item STAI), and 15.5 (sd = 5.5) for stress (6-item PSS).

As shown in Table 1, age, time in the US, years of schooling, days worked, and marital status did not differ by whether participants had experienced total nonpayment of due wages. The dichotomized not paid at all measure was associated with greater experiences of being paid less than promised (measured both categorically and continuously). Mean total scale scores for powerlessness, depression, anxiety, and stress did not differ by not being paid at all.

The dichotomized paid less than promised measure was not associated with age, time in the US, years of schooling, or days worked; however, it was not independent of marital status. Those who experienced underpayment of due wages were also more likely to experience total nonpayment. In contrast to not being paid at all, mean total scale scores for powerlessness, depression, anxiety, and stress were significantly higher among those who had experienced being paid less than promised.

### Primary Aim: Being Denied Earned Wages and Psychological Distress

The *total association* between each wage denial exposure (total nonpayment and underpayment of earned wages) and each indicator of psychological distress (depression, state anxiety, and stress) is shown in Table 2 (Unmediated Models 1 – 6). The coefficients correspond to path c in Figure 1A. In Unmediated Models 1 – 3, adjusted for sociodemographic characteristics, the frequency of **not being paid at all** in the past month was not significantly associated with depression, anxiety, nor stress. As shown in the Appendix, being formerly married, compared to having never been married, was associated with greater depression (b = 2.017, 95% CI [0.186, 4.164], p = .044); and the number of days worked was associated with lower anxiety (b =−0.085, 95% CI [−0.146, −0.021], p = .007). None of the sociodemographic variables were associated with stress.

In contrast, in similarly adjusted Unmediated Models 4 – 6, the frequency of **being paid less than promised** in the past month was significantly associated with each indicator of psychological distress: **depression** (b = 0.846, 95% CI [0.281, 1.298], p = .001); **anxiety** (b= 0.594, 95% CI [0.267, 0.905], p < .001); and stress (b = 1.021, 95% CI [0.378, 1.657], p < .002). Number of days worked was associated with lower anxiety (b = −0.100, 95% CI [−0.162, −0.039], p = .001. None of the sociodemographic variables were associated with depression or stress.

### Secondary Aim: Being Denied Due Wages and Psychological Distress via Perceived Powerlessness

#### Associations between Total Nonpayment, Powerlessness, and Psychological Distress

As shown in Table 3 (Mediation Models 1 – 3), number of in the past month was not associated with perceived powerlessness (path a in Figure 1B). In these three models, years of schooling (b =−0.153, 95% CI = −0.284, 0.024], p = .021) and number of days worked (b = −.081, 95% CI [−0.158, −0.002], p = .039 were both associated with decreased powerlessness.

Powerlessness, considered simultaneously with times not paid at all (path b in Figure 1B), was significantly associated with each indicator of psychological distress: (b = 0.554, 95% CI [0.425, 0.693], p = < .001); (b = 0.187, 95% CI [0.095, 0.285], p < .001); and (b = 0.523, 95% CI [0.363, 0.668], p < .001). However, there were no significant *indirect associations* (a*b) *direct associations* nor were there any (path c’ in Figure 1B) observed between times not paid at all and the psychological distress indicators. Thus, there was no evidence of a significant *mediated association* between experiencing nonpayment of due wages and depression, anxiety, nor stress. As shown in the Appendix, when considering nonpayment and powerlessness jointly, depression was not associated with any sociodemographic variable; however, days worked was associated with decreased anxiety (b =−0.070, 95% CI [−0.139, −0.008], p = .027), and years of schooling was associated with greater stress (b = 0.193, 95% CI [0.063, 0.318], p = .003).

#### Associations Between Underpayment, Powerlessness, and Psychological Distress

As shown in Table 4 (Mediation Models 4 – 6), **times paid less than promised** was significantly associated with powerlessness (path a) (b = 0.575, 95% CI [0.113, 1.077], p = 0.019). Years of schooling was associated with decreased powerlessness (b = −0.144, 95% CI [−0.274, −0.018], p= 0.031), as was days worked (b = −0.095, 95% CI [−0.177, −0.016], p= 0.015). Additionally, when considered simultaneously with underpayment, powerlessness was significantly associated with **depression** (path b in Figure 1B) (b = 0.531, 95% CI [0.389, 0.670], p< .001; **anxiety** (b = 0.167; CI [0.076, 0.271], p = .001), and **stress** (b = 0.493, 95% CI [0.335, 0.643], p < .001).

The *indirect association* (a*b) was significant between **times paid less than promised** and each indicator of psychological distress (**depression**: b = 0.305, 95% CI [0.055 – 0.586], p = .020; **anxiety**: b= 0.096, 95% CI [0.024– 0.224], p = 0.044; and **stress**: b = 0.283, 95% CI [0.056– 0.571], p = .023). As shown in the Appendix, depression was not associated with any sociodemographic variable when considering frequency of underpayment and powerlessness jointly; however, number of days worked was associated with decreased anxiety (b = −0.084, 95% CI [−0.150,−0.020], p = .008), and years of schooling was associated with increased stress (b = 0.198, 95% CI [0.066, 0.318], p = 0.003).

The *direct association* (path c’) was significant between **times paid less than promised** and each psychological distress outcome (**depression**: b = 0.541, 95% CI [0.083– 1.016], p = .020; **anxiety**: b= 0.498, 95% CI [0.203– 0.820], p = .001; and **stress**: b = 0.738, 95% CI [0.206– 1.264], p = .006), suggesting that the association between times paid less than promised and depression, anxiety, and stress may be *partially mediated* by powerlessness. The potential *mediated association* for each model is given by dividing the coefficient for the indirect association by the coefficient for the total association; thus, for **times paid less than promised**, 36.1% of its association with **depression**, 16.2% of its association with **anxiety**, and 27.7% of its association with **stress** may be explained by indirect pathways through perceived powerlessness.

### Synthesis: Comparison of Total, Direct, and Indirect Associations by Type of Wage Denial

As shown in Table 2 (Unmediated Models 1–6), the *total associations* (path c) between and each indicator of psychological distress (significant) were greater than those for (not significant). As shown in Tables 3 (Mediation Models 1–3: for nonpayment) and 4 (Mediation Models 4–6: for underpayment), associations between wage denial exposure and perceived powerlessness (path a) were greater for (significant) than for (not significant). In each mediation model, the association between powerlessness and the indicator of psychological distress, controlling for the wage denial exposure (path b) was higher for **total nonpayment** (significant) than for **underpayment** (significant). (a*b) for each indicator of psychological distress were higher for **underpayment** (significant for each outcome) than **for total nonpayment** (not significant for any outcome). *Direct associations* between **underpayment** and each indicator of psychological distress (path c’) were higher (and significant for each outcome) than those for **total nonpayment** (not significant for any outcome).

## Discussion

The main finding of this study was that a greater frequency of *being paid less than promised* (underpayment of due wages) in the past 30 days was significantly associated with increased levels of self-reported psychological distress, specifically depression, state anxiety, and perceived stress scores. Findings further indicated that the frequency of being paid less than promised may be indirectly associated with depression, state anxiety, and stress among LDLs through increased perceived powerlessness.

These findings align with the overall study hypotheses and provide quantitative support for prior qualitative research documenting the adverse mental health impacts of wage denial on Latino day laborers (LDLs), including experiences of depression [58], anxiety [34], feelings of worthlessness [58], and a sense of powerlessness due to fear of deportation and a perceived inability to seek justice [8, 27]. Additionally, our findings are consistent with evidence from the broader literature linking inadequate or unstable wages to adverse mental health outcomes [9–11, 39]. For example, research conceptualizing low wages as an occupational health hazard suggests that inadequate or unstable wages influence psychological distress through chronic financial strain, reduced perceived control, and persistent stress exposures [11]. Population-based studies indicate that experiences related to unpaid work in other labor contexts were associated with increased psychological distress and poorer self-rated health among immigrants and low-income workers, even outside the day labor context [9, 10]. Complementing this literature, quasi-experimental studies of minimum wage policy have demonstrated improvements in depressive symptoms and anxiety after the introduction of income increases among low-wage workers, underscoring the important role wages may play in shaping workers’ mental health [11, 39].

Notably, findings from the present study expand and clarify results from a previous study by the authors, which found no significant association between being denied due wages for completed work and depression, alcohol use, or social isolation [12]. However, as detailed in the previous study, the null finding may be attributable to methodological limitations addressed in the present study. Specifically, in the previous study, there was a lack of correspondence between being denied due wages (*on their last job*) and the indicators of mental health assessed (e.g., depression *in the last week*, alcohol use *in the past year*). Additionally, the previous study restricted LDL’s reports of being denied due wages to a single event/variable (i.e., both nonpayment and underpayment captured together with a binary yes/no measure, rather than disaggregated and continuous), which may have attenuated variability and our ability to detect an association with the mental health indicators assessed. By addressing these limitations, the current study provides a more robust assessment of the association between being denied due wages after completed work and mental health. Moreover, in the current analyses, associations between sociodemographic covariates and our indicators of psychological distress were generally modest and not consistent across outcomes; this pattern is consistent with the broader literature and further supports the robustness of the observed associations between wage denial and psychological distress.

An unexpected finding was that the frequency of *not being paid at all* (total nonpayment of due wages) was not significantly associated with depression, anxiety, nor stress. Given that total nonpayment is a more severe form of the two wage denial experiences assessed, we expected that it would have a similarly significant association as underpayment had with depression, anxiety, and stress. This null association may reflect a mismatch between the timeframe used to assess not being paid at all relative to the expected frequency of this experience. In our study, LDLs, on average, reported being paid less than promised twice as many times in the past 30 days than not being paid at all. We suspect that not being paid at all occurs less frequently than being paid less than promised, and using a one-month timeframe to assess not being paid at all may have been insufficient to capture this event. In addition, we hypothesize that despite being less severe (in absolute terms), the frequency with which LDLs experience being paid less than promised, relative to the more severe but less frequent experience of not being paid at all, may make being paid less than promised a recurrent stressor and thus more detrimental.

There are a few limitations to note in the current study. As it was cross-sectional, we cannot establish causality between the hypothesized predictors and the outcome variables. However, the purpose of the study was to provide an initial quantitative assessment of the association between being denied due wages for completed work and psychological distress among LDLs and explore the influence of perceived powerlessness on these associations as a potential mediator. We intended to explore the direction and magnitude of associations described rather than demonstrate causality. Second, the findings may not be generalizable to Latino day laborers broadly. This study focused only on LDLs looking for work in the Greater Houston metropolitan area in Texas, a context that may differ in important ways from other geographic locations where LDLs look for work.

The measures of wage denial, psychological distress (depression, state anxiety, stress), and powerlessness relied on self-reported data, making them vulnerable to recall bias, though this is minimized by assessing each for relatively brief time periods (i.e., experiences within the last day, week, month). We chose a 30-day window as the reference period for wage denial to maintain temporal alignment with at least one of our distress measures (stress, which uses a 30-day reference period), and to facilitate recall in this irregular work context. Notably, the short time frame to measure wage denial is also a measure not used previously, as it is generally assumed that it is a rare event, when in fact, it is a commonly reported experience among day laborers, especially being paid less than promised for a completed job. As with frequently experienced events, recall may be improved with a shorter reference period. Additionally, although perceived powerlessness accounted for a meaningful proportion of the association between the frequency of being paid less than promised and the psychological distress indicators, substantial unexplained pathways remain, suggesting that additional mechanisms may also contribute to these relationships. Lastly, statistical power may have been low for many of the associations assessed in the path models involving complete nonpayment due to the smaller effect sizes for most paths compared to those for underpayment. Issues of power are discussed more fully in MacKinnon et al. [66].

Despite these limitations, there are major strengths that make this study a significant contribution to the literature. To our knowledge, it is the first study to quantify the association of being denied due wages after a completed job with anxiety and stress. It is the second study to measure wage denial experiences, as a distinct variable from employer abuse broadly, and estimate its association with depression; furthermore, it improved upon methodological weaknesses in the first study that examined this association [12]. Moreover, this study is the first to investigate perceived powerlessness as a potential mediator of associations between being denied due wages and depression, anxiety, and stress.

Future studies using longitudinal data, larger sample sizes, and consideration of additional psychosocial mediators (e.g., financial strain, housing or food insecurity, fear of retaliation) are needed to validate and expand upon these results. Additionally, LDLs exposure to total nonpayment of due wages over a broader timeframe should be explored to capture this more severe but less frequent experience. Efforts to remediate nonpayment and underpayment of promised wages should include better enforcement of labor laws, a streamlined process for reporting labor law violations, legal assistance including protections for those who experience it and report it, and given the findings in this report, assistance to address the mental health impacts created or exacerbated by these experiences. The study's findings provide support that addressing the intentional failure to pay workers their due wages is crucial to promoting the overall well-being of LDLs.

## Supplementary Material

Appendix

**Supplementary Information** The online version contains supplementary material available at https://doi.org/10.1007/s40615-026-02972-6.

## Figures and Tables

**Table 1:** Characteristics of Latino day laborers within analytic sample (n= 274)

	Total Sample	Not Paid at All (total nonpayment of due wages)			Paid Less than Promised (underpayment of due wages)		
	
		No	Yes (≥1 times)	*p*-value	No	Yes (≥1 times)	*p*-value

	(*N* = 274)	(*N* = 234)			(*N* = 196)	(*N* = 78)	
**Sociodemographic Characteristics, mean (SD)**							
Age	45.2 (12.3)	45.1 (12.3)	45.4 (12.7)	0.989	44.8 (12.3)	46.1 (12.3)	0.733
Time in US	13.5 (12.2)	13.1 (11.9)	15.5 (13.7)	0.528	12.8 (11.3)	15.2 (14.0)	0.328
Years of School Completed	7.68 (4.47)	7.74 (4.45)	7.4 (4.6)	0.895	7.9 (4.3)	7.0 (4.9)	0.323
No. of Days Worked in the Last Month	8.29 (6.02	8.4 (6.1)	7.9 (5.6)	0.907	8.0 (6.2)	9.0 (5.5)	0.454
Marital Status, n (%)	-	-	-	0.988	-	-	0.006
Single, never married	133 (48.5%)	114 (48.7%)	19 (47.5%)	-	109 (55.6%)	24 (30.8%)	-
Married, living with a partner	101 (36.9%)	87 (37.2%)	14 (35.0%)	-	64 (32.7%)	37 (47.4%)	-
Divorced, widowed, or separated	40 (14.6%)	33 (14.1%)	7 (17.5%)	-	23 (11.7%)	17 (21.8%)	-
**Denied Due Wages for a Completed Job (No. of Times in the Last Month)**							
Times Not Paid at All, mean (SD)	0.3 (0.7)	-	-	-	0.1 (0.5)	0.6 (1.1)	< **0.001**
Times Not Paid at All, n (%)	-	-	-	-	-	-	**0.012**
None	234 (85.4%)	-	-	-	178 (90.8%)	56 (71.8%)	-
Once	22 (8.0%)	-	-	-	12 (6.1%)	10 (12.8%)	-
Twice	8 (2.9%)	-	-	-	2 (1.0%)	6 (7.7%)	-
Three times	6 (2.2%)	-	-	-	3 (1.5%)	3 (3.8%)	-
Four times	4 (1.5%)	-	-	-	1 (0.5%)	3 (3.8%)	-
Times Paid Less Than Promised, mean (SD)	0.6 (1.2)	0.5 (1.1)	1.4 (1.6)	< **0.001**	-	-	-
Times Paid Less than Promised, n (%)	-	-	-	**0.006**	-	-	-
None	196 (71.5%)	178 (76.1%)	18 (45.0%)	-	-	-	-
Once	28 (10.2%)	23 (9.8%)	5 (12.5%)	-	-	-	-
Twice	19 (6.9%)	14 (6.0%)	5 (12.5%)	-	-	-	-
Three times	16 (5.8%)	10 (4.3%)	6 (15.0%)	-	-	-	-
Four times	15 (5.5%)	9 (3.8%)	6 (15.0%)	-	-	-	-
**Perceived Powerlessness (Total Scale Scores), mean (SD)**	22.6 (4.3)	22.7 (4.2)	22.5 (4.6)	0.983	22.0 (4.2)	24.1 (4.0)	**0.001**
**Indicators of Psychological Distress (Total Scale Scores), mean (SD)**							
Depression	13.1 (4.9)	13.1 (5.0)	13.2 (4.7)	0.991	12.5 (4.8)	14.5 (4.9)	**0.008**
State Anxiety	10.5 (3.4)	10.4 (3.4)	11.3 (3.6)	0.363	10.2 (3.4)	11.5 (3.3)	**0.016**
Perceived Stress	15.5 (5.5)	15.4 (5.5)	16.1 (5.3)	0.771	14.9 (5.2)	17.1 (5.8)	**0.012**

a SD, standard deviation; Bolded p-values indicate *p* <.05, two-tailed

**Table 2:** Unmediated Models 1–6: Adjusted Path Coefficients for the Total Association between Being Denied Due Wages and Indicators of Psychological Distress among Latino Day Laborers (n= 274)

Denied due wage as “Not Paid at All"			Paid Less than Promised		
*Model 1: Association with Depression*					
	*Path*	*b*	*SE*	*95% CI*	*p*
Not Paid at All	*c*	0.283	0.548	−0.782–1.308	0.606
Model 2: Association with State Anxiety					
	*Path*	*b*	*SE*	*95% CI*	*p*
Not Paid at All	c	0.34	0.309	−0.219–1.029	0.272
Model 3: Association with Perceived Stress					
	*Path*	*b*	*SE*	*95% CI*	*p*
Not Paid at All	c	0.432	0.516	−0.689–1.325	0.402
**Denied due wage as “Paid less than Promised"**					
Model 4: Association with Depression					
	*Path*	*b*	*SE*	*95% CI*	*p*
	c	0.846	0.259	0.281–1.298	0.001

a The hypothesized conceptual model explored includes *Denied Due Wages (X)* as the independent variable; an *Indicator of Psychological Distress (Y)* as the dependent variable; and *Perceived Powerlessness (M)* as a potential mediator. Two levels of being *Denied Due Wages* were examined - the number of times in the last month that respondents were: (1) *Not Paid at All* and (2) *Paid Less than Promised* for a completed job. Three *Indicators of Psychological Distress* were examined: (1) *Depression;* (2) *State Anxiety;* and (3) *Perceived Stress.* Each level of wage denial was examined against each indicator of psychological distress in a separate model; thus, a total of six models were examined

b All models are adjusted for age in years, years in the US, years of school, days worked in the last month, and marital status

c b, path coefficient; SE, standard error; CI, confidence interval; Bolded p-values indicate *p* <.05, two-tailed

**Table 3:** Mediation Models 1–3: Adjusted Path Analysis Results for the Direct and Indirect Associations between Being Denied Due Wages Reported as Not Paid at All, Perceived Powerlessness, and Indicators of Psychological Distress among Latino Day Laborers (n= 274)

Association with Perceived Powerlessness					
*(estimate constant across Mediation Models 1 – 3 below)*					
	*Path*	*b*	*SE*	*95% CI*	*p*
Not Paid at All	a	0.097	0.463	−0.852 – 1.026	0.833
Mediation Model 1: Association with Depression					
	*Path*	*b*	*SE*	*95% CI*	*p*
Powerlessness	b	0.554	0.068	0.425 – 0.693	<0.001
*Direct Association*					
Not Paid at All	c’	0.229	0.407	−0.549 – 1.060	0.574
*Indirect Association*					
Not Paid at All	a*b	0.054	0.259	−0.460 – 0.569	0.835
Mediation Model 2: Association with State Anxiety					
	*Path*	*b*	*SE*	*95% CI*	*p*
Powerlessness	b	0.187	0.048	0.095 – 0.285	<0.001
*Direct Association*					
Not Paid at All	c’	0.322	0.298	−0.206 – 0.950	0.28
*Indirect Association*					
Not Paid at All	a*b	0.018	0.088	−0.149 – 0.199	0.836
Mediation Model 3: Association with Stress					
	*Path*	*b*	*SE*	*95% CI*	*p*
Powerlessness	b	0.523	0.078	0.363 – 0.668	<0.001
*Direct Association*					
Not Paid at All	c’	0.381	0.362	−0.418 – 1.010	0.293
*Indirect Association*					
Not Paid at All	a*b	0.051	0.247	−0.440 – 0.535	0.837

a The hypothesized conceptual model explored includes *Denied Due Wages (X)* as the independent variable; an *Indicator of Psychological Distress (Y)* as the dependent variable; and *Perceived Powerlessness (M)* as a potential mediator. Two levels of being *Denied Due Wages* were examined - the number of times in the last month that respondents were: (1) *Not Paid at All* and (2) *Paid Less than Promised* for a completed job. Three *Indicators of Psychological Distress* were examined: (1) *Depression;* (2) *State Anxiety;* and (3) *Perceived Stress.* Each level of wage denial was examined against each indicator of psychological distress in a separate model; thus, a total of six models were examined

b All models were adjusted for age, years in the US, years of school, days worked in the last month, and marital status

c b, path coefficient; SE, standard error; CI, confidence interval; Bolded p-values indicate *p* <.05, two-tailed

**Table 4:** Mediation Models 4–6: Adjusted Path Analysis Results for the Direct and Indirect Associations between Denied Wages Reported as Paid Less than Promised, Perceived Powerlessness, and Indicators of Psychological Distress among Latino Day Laborers (n= 290)

Association with Perceived Powerlessness					
*(estimate constant across Mediation Models 4 – 6 below)*					
	*Path*	*b*	*SE*	*95% CI*	*p*
Paid Less	a	0.575	0.244	0.113 – 1.077	0.019
Mediation Model 4: Association with Depression					
	*Path*	*b*	*SE*	*95% CI*	*p*
Powerlessness	b	0.531	0.07	0.389 – 0.670	< .001
*Direct Association*					
Paid Less	c’	0.541	0.232	0.083 – 1.016	.020
*Indirect Association*					
Paid Less	a*b	0.305	0.132	0.055 – 0.586	.020
Mediation Model 5: Association with State Anxiety					
	*Path*	*b*	*SE*	*95% CI*	*p*
Powerlessness	b	0.167	0.05	0.076 – 0.271	.001
*Direct Association*					
Paid Less	c’	0.498	0.156	0.203 – 0.820	.001
*Indirect Association*					
Paid Less	a*b	0.096	0.048	0.024 – 0.224	.044
Mediation Model 6: Association with Stress					
	*Path*	*b*	*SE*	*95% CI*	*p*
Powerlessness	b	0.493	0.078	0.335 – 0.643	< .001
*Direct Association*					
Paid Less	c’	0.738	0.269	0.206 – 1.264	.006
*Indirect Association*					
Paid Less	a*b	0.283	0.125	0.056 – 0.551	.023

a The hypothesized conceptual model explored includes *Denied Due Wages (X)* as the independent variable; an *Indicator of Psychological Distress (Y)* as the dependent variable; and *Perceived Powerlessness (M)* as a potential mediator. Two levels of being *Denied Due Wages* were examined - the number of times in the last month that respondents were: (1) *Not Paid at All* and (2) *Paid Less than Promised* for a completed job. Three *Indicators of Psychological Distress* were examined: (1) *Depression;* (2) *State Anxiety;* and (3) *Perceived Stress.* Each level of wage denial was examined against each indicator of psychological distress in a separate model; thus, a total of six models were examined

b All models were adjusted for age in years, years in the US, years of school, days worked in the last month, and marital status

c b, path coefficient; SE, standard error; CI, confidence interval; Bolded p-values indicate *p* <.05, two-tailed

## References

[1] Bobo K Wage Theft in America: Why Millions of Working Americans Are Not Getting Paid—And What We Can Do About It. New York: New; 2011. 10.2307/jj.32047644

[2] Cooper D, Kroeger T. Employers steal billions from workers’ paychecks each year. Washington, D.C.: Economic Policy Institute; 2017.

[3] Minkler M, Wage Theft as a Neglected Public Health Problem: An Overview and Case Study From San Francisco’s Chinatown District. Am J Public Health. 2014;104(6):1010–20. 24825200 10.2105/AJPH.2013.301813 PMC4062017

[4] Bernhardt A, Broken laws, unprotected workers: Violations of employment and labor laws in America’s cities. UCLA Institute for Research on Labor and Employment: Los Angeles; 2009.

[5] Mangundayao I, More than $3 billion in stolen wages recovered for workers between 2017 and 2020. Washington, D.C.: Economic Policy Institute; 2021.

[6] Gleeson S Precarious claims. University of California Press; 2016.

[7] Salinas JL, Salinas M. Systemic racism and undocumented Latino migrant laborers during COVID-19: A narrative review and implications for improving occupational health. J Migration Health. 2022;5:100106. 35434678 10.1016/j.jmh.2022.100106 PMC9004221

[8] Fussell E The deportation threat dynamic and victimization of Latino migrants: wage theft and robbery. Sociol Quart. 2011;52(4):593–615.

[9] Eisenberg-Guyot J, Wage theft and life expectancy inequities in the United States: A simulation study. Prev Med. 2022;159:107068. 35469776 10.1016/j.ypmed.2022.107068 PMC9246227

[10] Lee KF, Nakphong MK, Young M-EDT. The legacy of immigration policies and employment exclusion: Assessing the relationship between employment exclusions and immigrant health. SSM - Popul Health. 2024;26:101676. 38711566 10.1016/j.ssmph.2024.101676 PMC11070755

[11] Leigh JP, Vogli RD. Low Wages as Occupational Health Hazards. J Occup Environ Med. 2016;58(5):444–7. 27158950 10.1097/JOM.0000000000000717

[12] Fernández-Esquer ME, Structural racism and immigrant health: exploring the association between wage theft, mental health, and injury among latino day laborers. Ethnicity & Disease, 2021. 31(Suppl 1): p. 345–356. 10.18865/ed.31.S1.345 34045836 PMC8143848

[13] Bailey ZD, Feldman JM, Bassett MT. How structural racism works — racist policies as a root cause of U.S. racial health inequities. N Engl J Med. 2021;384(8):768–73. 33326717 10.1056/NEJMms2025396 PMC11393777

[14] Bailey ZD, Structural racism and health inequities in the USA: evidence and interventions. Lancet. 2017;389(10077):1453–63. 28402827 10.1016/S0140-6736(17)30569-X

[15] Braveman PA, Systemic And Structural Racism: Definitions, Examples, Health Damages, And Approaches To Dismantling. Health Aff. 2022;41(2):171–8. 10.1377/hlthaff.2021.01394 35130057

[16] Galemba RB. They steal our work: wage theft and the criminalization of immigrant day laborers in Colorado, USA. Eur J Crim Policy Res. 2021;27(1):91–112.

[17] Quesada J, Hart LK, Bourgois P. Structural vulnerability and health: Latino migrant laborers in the United States. Med Anthropol. 2011;30(4):339–62. 21777121 10.1080/01459740.2011.576725 PMC3146033

[18] Lee S Policing wage theft in the day labor market. UC Irvine Law Rev. 2014;4(2):655.

[19] Ruan N Facilitating wage theft: how courts use procedural rules to undermine substantive rights of low-wage workers. Vand L Rev. 2010;63(3):725.

[20] Siqueira CE, Effects of social, economic, and labor policies on occupational health disparities. AM J IND MED. 2014;57(5):557–72. 23606055 10.1002/ajim.22186 PMC5920651

[21] Stuesse A When they’re done with you: legal violence and structural vulnerability among injured immigrant poultry workers. Anthropol Work Rev. 2018;39(2):79–93.

[22] Valenzuela A, On the corner: Day labor in the United States. UCLA Center for the Study of Urban Poverty; 2006.

[23] Quesada J, “As Good As It Gets”: Undocumented Latino Day Laborers Negotiating Discrimination in San Francisco and Berkeley, California, USA. City & Society. 2014;26(1):29–50. 24910501 10.1111/ciso.12033 PMC4043379

[24] Hill CM, Williams EC, Ornelas IJ. Help Wanted: Mental Health and Social Stressors Among Latino Day Laborers. Am J Men’s Health. 2019;13(2):1557988319838424. 30880547 10.1177/1557988319838424 PMC6438433

[25] Valenzuela A Day labor work. Annu Rev Sociol. 2003;29(1):307–33.

[26] Haro AY, Beyond Occupational Hazards: Abuse of Day Laborers and Health. J Immigr Minor Health. 2020;22(6):1172–83. 32989653 10.1007/s10903-020-01094-3

[27] Negi NJ. Battling discrimination and social isolation: psychological distress among Latino day laborers. Am J Community Psychol. 2013;51(1–2):164–74. 22864958 10.1007/s10464-012-9548-0

[28] Negi NJ, They dumped me like trash”: the social and psychological toll of victimization on Latino day laborers’ lives. Am J Community Psychol. 2020;65(3–4):369–80. 31821570 10.1002/ajcp.12406 PMC10044443

[29] Waren W Wage theft among Latino day laborers in post-Katrina New Orleans: comparing contractors with other employers. J Int Migr Integr. 2014;15(4):737–51.

[30] Bauer M, Reynolds S. Under Siege: Life for Low-income Latinos in the South: a Report by the Southern Poverty Law Center. Southern Poverty Law Center; 2009.

[31] Sung H-E, Tyrannizing Strangers for Profit: Wage Theft, Cross-Border Migrant Workers, and the Politics of Exclusion in an Era of Global Economic Integration. Outside Justice: Immigration and the Criminalizing Impact of Changing Policy and Practice. New York: New York, NY: Springer; 2013. pp. 247–67. Brotherton DC, Stageman DL, and Leyro SP, Editors.

[32] Lee JJ, Smith A. Regulating wage theft. Wash L Rev. 2019;94:759.

[33] Milkman R, González AL, Narro V. Wage theft and workplace violations in Los Angeles: The failure of employment and labor law for low-wage workers. UCLA Institute for Research on Labor and Employment: Los Angeles; 2010.

[34] Negi NJ. Identifying psychosocial stressors of well-being and factors related to substance use among Latino day laborers. J Immigr Minor Health. 2011;13(4):748–55. 21107694 10.1007/s10903-010-9413-x PMC3523724

[35] Sanidad C Stories from Immigrant Workers in the Valley of the Sun: Status, Wage Theft, Recourse, and Resilience. Arizona State University; 2011.

[36] Organista KC, Living Conditions and Psychological Distress in Latino Migrant Day Laborers: The Role of Cultural and Community Protective Factors. Am J Community Psychol. 2017;59(1–2):94–105. 27996094 10.1002/ajcp.12113

[37] Prins SJ, The Serpent of Their Agonies. Epidemiology. 2021;32(2):303–9. 33252438 10.1097/EDE.0000000000001304 PMC7872213

[38] Leigh JP. Treatment design, health outcomes, and demographic categories in the literature on minimum wages and health. Econ Hum Biology. 2021;43:101043. 10.1016/j.ehb.2021.101043 34425521

[39] Reeves A, Introduction of a National Minimum Wage Reduced Depressive Symptoms in Low-Wage Workers: A Quasi-Natural Experiment in the UK. Health Econ. 2017;26(5):639–55. 27046821 10.1002/hec.3336 PMC5396382

[40] Bryant-Davis T Healing requires recognition: The case for race-based traumatic stress. Couns Psychol. 2007;35(1):135–43.

[41] Chavez-Dueñas NY, Healing ethno-racial trauma in Latinx immigrant communities: Cultivating hope, resistance, and action. Am Psychol. 2019;74:49–62. 30652899 10.1037/amp0000289

[42] Lockwood S, Cuevas CA. Hate Crimes and Race-Based Trauma on Latinx Populations: A Critical Review of the Current Research. Trauma, Violence, & Abuse. 2022;23(3):854–67. 10.1177/1524838020979688 33325321

[43] Bryant-Davis T, Ocampo C. Racist incident–based trauma. Couns Psychol. 2005;33(4):479–500.

[44] Loo CM, Race-related stress among Asian American veterans: A model to enhance diagnosis and treatment. Cult Divers Mental Health. 1998;4(2):75–90. 9586340

[45] Hwang W-C, Goto S The impact of perceived racial discrimination on the mental health of Asian American and Latino college students. Cult Divers Ethnic Minor Psychol. 2008;14(4):326. 10.1037/1099-9809.14.4.326 18954168

[46] Andrade N, Ford AD, Alvarez C. Discrimination and Latino health: a systematic review of risk and resilience. Hispan Health Care Int. 2021;19(1):5–16. 10.1177/1540415320921489 32380912

[47] Goto JB, Couto PFM, Bastos JL. Systematic review of epidemiological studies on interpersonal discrimination and mental health. Cad Saude Publica. 2013;29:445–59. 23532281 10.1590/s0102-311x2013000300004

[48] Williams DR, Mohammed SA. Discrimination and racial disparities in health: evidence and needed research. J Behav Med. 2009;32(1):20–47. 19030981 10.1007/s10865-008-9185-0 PMC2821669

[49] Williams DR, Neighbors HW, Jackson JS. Racial/ethnic discrimination and health: findings from community studies. Am J Public Health. 2003;93(2):200–8. 12554570 10.2105/ajph.93.2.200 PMC1447717

[50] Flores E, Perceived Discrimination, Perceived Stress, and Mental and Physical Health Among Mexican-Origin Adults. Hispanic J Behav Sci. 2008;30(4):401–24.

[51] Walter N, Bourgois P, Loinaz HM. Masculinity and undocumented labor migration: injured latino day laborers in San Francisco. Social Science & Medicine. 2004;59(6):1159–68. 15210088 10.1016/j.socscimed.2003.12.013 PMC2690638

[52] Organista KC, Kubo A. Pilot survey of HIV risk and contextual problems and issues in Mexican/Latino migrant day laborers. J Immigr Minor Health. 2005;7(4):269–81. 10.1007/s10903-005-5124-0 19813293

[53] Galvan FH, Chronic Stress Among Latino Day Laborers. Hispanic J Behav Sci. 2015;37(1):75–89.

[54] Duke MR, Bourdeau B, Hovey JD. Day laborers and occupational stress: testing the migrant stress inventory with a Latino day laborer population. Cult Divers Ethnic Minor Psychol. 2010;16(2):116. 10.1037/a0018665 20438149

[55] Fernández-Esquer ME, Gallardo KR, Diamond PM. Predicting the Influence of Situational and Immigration Stress on Latino Day Laborers’ Workplace Injuries: An Exploratory Structural Equation Model. J Immigr Minor Health. 2019;21(2):364–71. 29767403 10.1007/s10903-018-0752-3

[56] Nelson RW Jr, Sociodemographic characteristics, health, and success at obtaining work among Latino urban day laborers. J Health Care Poor Underserved. 2012;23(2):797–810. 22643625 10.1353/hpu.2012.0041

[57] de Castro AB, Stressors among Latino Day Laborers:A Pilot Study Examining Allostatic Load. AAOHN J. 2010;58(5):185–96. 20507008 10.3928/08910162-20100428-01 PMC2964275

[58] Walter N, Social context of work injury among undocumented day laborers in San Francisco. J GEN INTERN MED. 2002;17(3):221–9. 11929509 10.1046/j.1525-1497.2002.10501.x PMC1495019

[59] Daniel-Ulloa J, Sun C, Rhodes SD. The intersection between masculinity and health among rural immigrant Latino men. Int J Mens Health. 2017;16(1):84–95. 29422781 PMC5798237

[60] Organista KC, Sexual health of Latino migrant day labourers under conditions of structural vulnerability. Cult Health Sex. 2013;15(1):58–72. 23140484 10.1080/13691058.2012.740075 PMC3743724

[61] Lim S, Exploratory and confirmatory factor analyses and invariance assessment of the perceived powerlessness scale among youth in Baltimore. J Health Psychol. 2020;25(10–11):1644–56. 29637800 10.1177/1359105318769349 PMC6119533

[62] Zhu Y, Psychological distress and metabolomic markers: A systematic review of posttraumatic stress disorder, anxiety, and subclinical distress. Neurosci Biobehavioral Reviews. 2022;143:104954. 10.1016/j.neubiorev.2022.104954 PMC9729460 36368524

[63] Levine SZ. Evaluating the seven-item Center for Epidemiologic Studies Depression Scale short-form: a longitudinal US community study. Social Psychiatry and Psychiatric Epidemiology. 2013;48(9):1519–26. 23299927 10.1007/s00127-012-0650-2

[64] Perpiñá-Galvañ J, Cabañero-Martínez MJ, Richart-Martínez M. Reliability and validity of shortened State Trait Anxiety Inventory in Spanish patients receiving mechanical ventilation. Am J Crit Care. 2013;22(1):46–52. 23283088 10.4037/ajcc2013282

[65] Cohen S, Kamarck T, Mermelstein R. A global measure of perceived stress. J Health Soc Behav. 1983;24(4):385–96. 6668417

[66] MacKinnon DP, Fairchild AJ, Fritz MS. Mediation Anal Annual Rev Psychol. 2007;58(1):593–614. 10.1146/annurev.psych.58.110405.085542 PMC2819368 16968208

[67] Rosseel Y. lavaan: AnRPackage for Structural Equation Modeling. J Stat Softw, 2012. 48(2).

