# Supplementary material for: Denial of Earned Wages and Psychological Distress among Latino Day Laborers: The Impact of Work-Related Mistreatment and Powerlessness on Mental Health
Source: J Racial Ethn Health Disparities. Author manuscript; Available in PMC 2026 Sep 1. (PMC13532408; doi:10.1007/s40615-026-02972-6)
Supplement: Supplementary file 1 [file NIHMS2203887-supplement-Appendix.docx]

**APPENDIX. Full Model Results (including Sociodemographic Covariates): Adjusted Path Analysis for Total, Direct, and Indirect Associations for Models 1 – 6**

Model 1. Full Model Results (including Covariates): Adjusted Path Analysis for Total, Direct, and Indirect Associations between Being Denied Due Wages Reported as Not Paid at All (Total Nonpayment), Perceived Powerlessness, and Depression among Latino Day Laborers, (n=274)

|  | ***b*** | ***SE*** | ***95% CI*** | ***p*** |
| --- | --- | --- | --- | --- |
| **Not Paid at all and Depression** |  |  |  |  |
| Not Paid at all **(total effect, path c)** | 0.283 | 0.548 | [-0.782, 1.308] | 0.606 |
| Years in US | 0 | 0.027 | [-0.054, 0.053] | 0.993 |
| Years of School | -0.1 | 0.07 | [-0.241, 0.028] | 0.152 |
| Age | -0.025 | 0.028 | [-0.078, 0.028] | 0.373 |
| Marital Status |  |  |  |  |
| Never Married (referral category) | -- | -- | -- | -- |
| Married or Living with a Partner | 0.787 | 0.668 | [-0.490, 2.126] | 0.239 |
| Formerly Married | 2.017 | 1.003 | [0.186, 4.164] | **0.044** |
| Days Worked Last 30 Days | -0.029 | 0.061 | [-0.147, 0.099] | 0.631 |
|  |  |  |  |  |
| **Not Paid at all and Powerlessness** |  |  |  |  |
| Not Paid at all **(path a)** | 0.097 | 0.463 | [-0.852, 1.026] | 0.833 |
| Years in US | -0.026 | 0.022 | [-0.068, 0.016] | 0.225 |
| Years of School | -0.153 | 0.066 | [-0.284, -0.024] | **0.021** |
| Age | 0.034 | 0.025 | [-0.017, 0.081] | 0.165 |
| Marital Status |  |  |  |  |
| Never Married (referral category) | -- | -- | -- | -- |
| Married or Living with a Partner | 0.458 | 0.51 | [-0.449, 1.605] | 0.37 |
| Formerly Married | 1.007 | 0.931 | [-0.819, 2.942] | 0.279 |
| Days Worked Last 30 Days | -0.081 | 0.039 | [-0.158, -0.002] | **0.039** |
|  |  |  |  |  |
| **Not Paid at all, Powerlessness and Depression** |  |  |  |  |
| Not Paid at all **(direct effect, path c’)** | 0.229 | 0.407 | [-0.549, 1.060] | 0.574 |
| Powerlessness **(path b)** | 0.554 | 0.068 | [0.425, 0.693] | **<.001** |
| Years in US | 0.014 | 0.023 | [-0.031, 0.064] | 0.54 |
| Years of School | -0.015 | 0.057 | [-0.137, 0.088] | 0.793 |
| Age | -0.044 | 0.025 | [-0.089, 0.005] | 0.077 |
| Marital Status |  |  |  |  |
| Never Married (referral category) | -- | -- | -- | -- |
| Married or Living with a Partner | 0.534 | 0.61 | [-0.634, 1.802] | 0.382 |
| Formerly Married | 1.46 | 0.83 | [0.006, 3.222] | 0.079 |
| Days Worked Last 30 Days | 0.016 | 0.054 | [-0.089, 0.120] | 0.773 |

^a^ b, coefficient; SE, standard error; CI, confidence interval. Bolded p-values indicate p < .05, two-tailed.

Model 2. Full Model Results (including Covariates): Adjusted Path Analysis for Total, Direct, and Indirect Associations between Being Denied Due Wages Reported as Not Paid at All (Total Nonpayment), Perceived Powerlessness, and State Anxiety among Latino Day Laborers, (n=274)

|  | ***b*** | ***SE*** | ***95% CI*** | ***p*** |
| --- | --- | --- | --- | --- |
| **Total Effect: Not Paid at All and Anxiety** |  |  |  |  |
| Not Paid at All **(total effect, path c)** | 0.34 | 0.309 | [-0.219, 1.029] | 0.272 |
| Years in US | -0.012 | 0.021 | [-0.050, 0.029] | 0.555 |
| Years of School | 0.049 | 0.049 | [-0.051, 0.138] | 0.313 |
| Age | 0.012 | 0.019 | [-0.027, 0.048] | 0.525 |
| Marital Status |  |  |  |  |
| Never Married (referral category) | -- | -- | -- | -- |
| Married or Living with a Partner | 0.46 | 0.453 | [-0.478, 1.368] | 0.31 |
| Formerly Married | 0.015 | 0.699 | [-1.219, 1.530] | 0.983 |
| Days Worked Last 30 Days | -0.085 | 0.032 | [-0.146, -0.021] | **0.007** |
|  |  |  |  |  |
| **Not Paid at All and Powerlessness** |  |  |  |  |
| Not Paid at All **(path a)** | 0.097 | 0.463 | [-0.852, 1.026] | 0.833 |
| Years in US | -0.026 | 0.022 | [-0.068, 0.016] | 0.225 |
| Years of School | -0.153 | 0.066 | [-0.284, -0.024] | **0.021** |
| Age | 0.034 | 0.025 | [-0.017, 0.081] | 0.165 |
| Marital Status |  |  |  |  |
| Never Married (referral category) | -- | -- | -- | -- |
| Married or Living with a Partner | 0.458 | 0.51 | [-0.449, 1.605] | 0.37 |
| Formerly Married | 1.007 | 0.931 | [-0.819, 2.942] | 0.279 |
| Days Worked Last 30 Days | -0.081 | 0.039 | [-0.158, -0.002] | **0.039** |
|  |  |  |  |  |
| **Not Paid at All, Powerlessness and Anxiety** |  |  |  |  |
| Not Paid at All **(direct effect, path c’)** | 0.322 | 0.298 | [-0.206, 0.950] | 0.28 |
| Powerlessness **(path b)** | 0.187 | 0.048 | [0.095, 0.285] | **<.001** |
| Years in US | -0.008 | 0.02 | [-0.045, 0.033] | 0.701 |
| Years of School | 0.078 | 0.044 | [-0.005, 0.169] | 0.076 |
| Age | 0.006 | 0.018 | [-0.033, 0.040] | 0.764 |
| Marital Status |  |  |  |  |
| Never Married (referral category) | -- | -- | -- | -- |
| Married or Living with a Partner | 0.374 | 0.466 | [-0.585, 1.203] | 0.421 |
| Formerly Married | -0.173 | 0.643 | [-1.327, 1.191] | 0.788 |
| Days Worked Last 30 Days | -0.07 | 0.032 | [-0.139, -0.008] | **0.027** |

^a^ b, coefficient; SE, standard error; CI, confidence interval. Bolded p-values indicate p < .05, two-tailed.

Model 3. Full Model Results (including Covariates): Adjusted Path Analysis for Total, Direct, and Indirect Associations between Being Denied Due Wages Reported as Not Paid at All (Total Nonpayment), Perceived Powerlessness, and Perceived Stress among Latino Day Laborers, (n=274)

|  | ***b*** | ***SE*** | ***95% CI*** | ***p*** |
| --- | --- | --- | --- | --- |
| **Not Paid at All and Stress** |  |  |  |  |
| Not Paid at All **(total effect, path c)** | 0.432 | 0.516 | [−0.689, 1.325] | 0.402 |
| Years in US | −0.002 | 0.033 | [−0.062, 0.072] | 0.956 |
| Years of School | 0.113 | 0.074 | [−0.022, 0.276] | 0.13 |
| Age | 0.016 | 0.031 | [−0.042, 0.078] | 0.611 |
| Marital Status |  |  |  |  |
| Never Married (referral category) | -- | -- | -- | -- |
| Married or Living with a Partner | 0.905 | 0.715 | [−0.480, 2.372] | 0.206 |
| Formerly Married | 1.424 | 0.978 | [−0.469, 3.433] | 0.145 |
| Days Worked Last 30 Days | −0.093 | 0.066 | [−0.223, 0.031] | 0.161 |
|  |  |  |  |  |
| **Not Paid at All and Powerlessness** |  |  |  |  |
| Not Paid at All **(path a)** | 0.097 | 0.463 | [−0.852, 1.026] | 0.833 |
| Years in US | −0.026 | 0.022 | [−0.068, 0.016] | 0.225 |
| Years of School | −0.153 | 0.066 | [−0.284, −0.024] | **0.021** |
| Age | 0.034 | 0.025 | [−0.017, 0.081] | 0.165 |
| Marital Status |  |  |  |  |
| Never Married (referral category) | -- | -- | -- | -- |
| Married or Living with a Partner | 0.458 | 0.51 | [−0.449, 1.605] | 0.37 |
| Formerly Married | 1.007 | 0.931 | [−0.819, 2.942] | 0.279 |
| Days Worked Last 30 Days | −0.081 | 0.039 | [−0.158, −0.002] | **0.039** |
|  |  |  |  |  |
| **Not Paid at All, Powerlessness and Stress** |  |  |  |  |
| Not Paid at All **(direct effect, path c’)** | 0.381 | 0.362 | [−0.418, 1.010] | 0.293 |
| Powerlessness **(path b)** | 0.523 | 0.078 | [0.363, 0.668] | **<.001** |
| Years in US | 0.012 | 0.031 | [−0.053, 0.069] | 0.696 |
| Years of School | 0.193 | 0.066 | [0.063, 0.318] | **0.003** |
| Age | −0.002 | 0.029 | [−0.059, 0.063] | 0.941 |
| Marital Status |  |  |  |  |
| Never Married (referral category) | -- | -- | -- | -- |
| Married or Living with a Partner | 0.666 | 0.676 | [−0.731, 1.929] | 0.324 |
| Formerly Married | 0.898 | 0.881 | [−0.866, 2.573] | 0.308 |
| Days Worked Last 30 Days | −0.051 | 0.062 | [−0.176, 0.070] | 0.415 |

^a^ b, coefficient; SE, standard error; CI, confidence interval. Bolded p-values indicate p < .05, two-tailed.

Model 4. Full Model Results (including Covariates): Adjusted Path Analysis for Total, Direct, and Indirect Associations between Being Denied Due Wages Reported as Paid Less than Promised (Underpayment), Perceived Powerlessness, and Depression among Latino Day Laborers, (n=274)

|  | ***b*** | ***SE*** | ***95% CI*** | ***p*** |
| --- | --- | --- | --- | --- |
| **Paid Less and Depression** |  |  |  |  |
| Paid Less **(total effect, path c)** | 0.846 | 0.259 | [0.281, 1.298] | **0.001** |
| Years in US | -0.004 | 0.026 | [-0.053, 0.048] | 0.889 |
| Years of School | -0.087 | 0.069 | [-0.228, 0.035] | 0.209 |
| Age | -0.022 | 0.027 | [-0.075, 0.030] | 0.418 |
| Marital Status |  |  |  |  |
| Never Married (referral category) | -- | -- | -- | -- |
| Married or Living with a Partner | 0.299 | 0.664 | [-0.973, 1.612] | 0.652 |
| Formerly Married | 1.506 | 1.01 | [-0.365, 3.592] | 0.136 |
| Days Worked Last 30 Days | -0.05 | 0.062 | [-0.170, 0.076] | 0.423 |
|  |  |  |  |  |
| **Paid Less and Powerlessness** |  |  |  |  |
| Paid Less **(path a)** | 0.575 | 0.244 | [0.113, 1.077] | **0.019** |
| Years in US | -0.029 | 0.021 | [-0.072, 0.012] | 0.171 |
| Years of School | -0.144 | 0.067 | [-0.274, -0.018] | **0.031** |
| Age | 0.036 | 0.024 | [-0.014, 0.084] | 0.14 |
| Marital Status |  |  |  |  |
| Never Married (referral category) | -- | -- | -- | -- |
| Married or Living with a Partner | 0.12 | 0.514 | [-0.775, 1.249] | 0.816 |
| Formerly Married | 0.655 | 0.916 | [-1.173, 2.567] | 0.474 |
| Days Worked Last 30 Days | -0.095 | 0.039 | [-0.177, -0.016] | **0.015** |
|  |  |  |  |  |
| **Stress, Powerlessness and Depression** |  |  |  |  |
| Paid Less **(direct effect, path c’)** | 0.541 | 0.232 | [0.083, 1.016] | **0.02** |
| Powerlessness **(path b)** | 0.531 | 0.07 | [0.389, 0.670] | **<.001** |
| Years in US | 0.012 | 0.023 | [-0.035, 0.058] | 0.603 |
| Years of School | -0.011 | 0.058 | [-0.132, 0.096] | 0.852 |
| Age | -0.041 | 0.025 | [-0.088, 0.008] | 0.092 |
| Marital Status |  |  |  |  |
| Never Married (referral category) | -- | -- | -- | -- |
| Married or Living with a Partner | 0.236 | 0.615 | [-0.936, 1.499] | 0.701 |
| Formerly Married | 1.158 | 0.85 | [-0.379, 2.982] | 0.173 |
| Days Worked Last 30 Days | 0.001 | 0.055 | [-0.109, 0.107] | 0.989 |

^a^ b, coefficient; SE, standard error; CI, confidence interval. Bolded p-values indicate p < .05, two-tailed.

Model 5. Full Model Results (including Covariates): Adjusted Path Analysis for Total, Direct, and Indirect Associations between Being Denied Due Wages Reported as Paid Less than Promised (Underpayment), Perceived Powerlessness, and State Anxiety among Latino Day Laborers, (n=274)

|  | ***b*** | ***SE*** | ***95% CI*** | ***p*** |
| --- | --- | --- | --- | --- |
| **Paid Less and Anxiety** |  |  |  |  |
| Paid Less **(total effect, path c)** | 0.594 | 0.161 | [0.267, 0.905] | **<.001** |
| Years in US | -0.014 | 0.021 | [-0.053, 0.030] | 0.514 |
| Years of School | 0.057 | 0.048 | [-0.044, 0.145] | 0.236 |
| Age | 0.013 | 0.018 | [-0.024, 0.049] | 0.463 |
| Marital Status |  |  |  |  |
| Never Married (referral category) | -- | -- | -- | -- |
| Married or Living with a Partner | 0.128 | 0.468 | [-0.814, 1.038] | 0.785 |
| Formerly Married | -0.337 | 0.7 | [-1.628, 1.213] | 0.63 |
| Days Worked Last 30 Days | -0.1 | 0.031 | [-0.162, -0.039] | **0.001** |
|  |  |  |  |  |
| **Paid Less and Powerlessness** |  |  |  |  |
| Paid Less **(path a)** | 0.575 | 0.244 | [0.113, 1.077] | **0.019** |
| Years in US | -0.029 | 0.021 | [-0.072, 0.012] | 0.171 |
| Years of School | -0.144 | 0.067 | [-0.274, -0.018] | **0.031** |
| Age | 0.036 | 0.024 | [-0.014, 0.084] | 0.14 |
| Marital Status |  |  |  |  |
| Never Married (referral category) | -- | -- | -- | -- |
| Married or Living with a Partner | 0.12 | 0.514 | [-0.775, 1.249] | 0.816 |
| Formerly Married | 0.655 | 0.916 | [-1.173, 2.567] | 0.474 |
| Days Worked Last 30 Days | -0.095 | 0.039 | [-0.177, -0.016] | **0.015** |
|  |  |  |  |  |
| **Paid Less, Powerlessness and Anxiety** |  |  |  |  |
| Paid Less **(direct effect, path c’)** | 0.498 | 0.156 | [0.203, 0.820] | **0.001** |
| Powerlessness **(path b)** | 0.167 | 0.05 | [0.076, 0.271] | **0.001** |
| Years in US | -0.009 | 0.02 | [-0.047, 0.031] | 0.654 |
| Years of School | 0.081 | 0.044 | [0.000, 0.175] | 0.064 |
| Age | 0.007 | 0.018 | [-0.031, 0.041] | 0.681 |
| Marital Status |  |  |  |  |
| Never Married (referral category) | -- | -- | -- | -- |
| Married or Living with a Partner | 0.108 | 0.48 | [-0.876, 0.936] | 0.822 |
| Formerly Married | -0.446 | 0.647 | [-1.679, 0.904] | 0.490 |
| Days Worked Last 30 Days | -0.084 | 0.032 | [-0.150, -0.020] | **0.008** |

^a^ b, coefficient; SE, standard error; CI, confidence interval. Bolded p-values indicate p < .05, two-tailed.

Model 6. Full Model Results (including Covariates): Adjusted Path Analysis for Total, Direct, and Indirect Associations between Being Denied Due Wages Reported as Paid Less than Promised (Underpayment), Perceived Powerlessness, and Perceived Stress among Latino Day Laborers, (n=274)

|  | ***b*** | ***SE*** | ***95% CI*** | ***p*** |
| --- | --- | --- | --- | --- |
| **Paid Less and Stress** |  |  |  |  |
| Paid Less **(total effect, path c)** | 1.021 | 0.328 | [0.378, 1.657] | **0.002** |
| Years in US | −0.005 | 0.031 | [−0.061, 0.064] | 0.868 |
| Years of School | 0.127 | 0.074 | [−0.018, 0.273] | 0.085 |
| Age | 0.019 | 0.031 | [−0.039, 0.078] | 0.546 |
| Marital Status |  |  |  |  |
| Never Married (referral category) | -- | -- | -- | -- |
| Married or Living with a Partner | 0.324 | 0.716 | [−1.059, 1.869] | 0.651 |
| Formerly Married | 0.812 | 0.972 | [−1.004, 2.781] | 0.404 |
| Days Worked Last 30 Days | −0.117 | 0.065 | [−0.238, 0.013] | 0.072 |
|  |  |  |  |  |
| **Paid Less and Powerlessness** |  |  |  |  |
| Paid Less **(path a)** | 0.575 | 0.244 | [0.113, 1.077] | **0.019** |
| Years in US | −0.029 | 0.021 | [−0.072, 0.012] | 0.171 |
| Years of School | −0.144 | 0.067 | [−0.274, −0.018] | **0.031** |
| Age | 0.036 | 0.024 | [−0.014, 0.084] | 0.14 |
| Marital Status |  |  |  |  |
| Never Married (referral category) | -- | -- | -- | -- |
| Married or Living with a Partner | 0.120 | 0.514 | [−0.775, 1.249] | 0.816 |
| Formerly Married | 0.655 | 0.916 | [−1.173, 2.567] | 0.474 |
| Days Worked Last 30 Days | −0.095 | 0.039 | [−0.177, −0.016] | **0.015** |
|  |  |  |  |  |
| **Paid Less, Powerlessness and Stress** |  |  |  |  |
| Paid Less **(direct effect, path c’)** | 0.738 | 0.269 | [0.206, 1.264] | **0.006** |
| Powerlessness **(path b)** | 0.493 | 0.078 | [0.335, 0.643] | **<.001** |
| Years in US | 0.009 | 0.029 | [−0.054, 0.061] | 0.749 |
| Years of School | 0.198 | 0.066 | [0.066, 0.318] | **0.003** |
| Age | 0.001 | 0.029 | [−0.058, 0.062] | 0.975 |
| Marital Status |  |  |  |  |
| Never Married (referral category) | -- | -- | -- | -- |
| Married or Living with a Partner | 0.265 | 0.684 | [−1.17, 1.517] | 0.699 |
| Formerly Married | 0.489 | 0.885 | [−1.317, 2.174] | 0.58 |
| Days Worked Last 30 Days | −0.071 | 0.062 | [−0.19, 0.054] | 0.255 |

^a^ b, coefficient; SE, standard error; CI, confidence interval. Bolded p-values indicate p < .05, two-tailed.
